# ORAL HEALTH EQUITY, BELIEFS, PRACTICES, PERCEPTIONS AMONG OLDER DETROIT RESIDENTS

**DOI:** 10.1093/geroni/igad104.1646

**Published:** 2023-12-21

**Authors:** Mark Luborsky, Jocie Osika, Kimberly Shay, Mariam Shilbayeh, Anisa Anisa Simoni, Candace Ziglor, Divesh Byrappagari, Judith Jones

**Affiliations:** Wayne State University, Detroit, Michigan, United States; Wayne State University, Detroit, Michigan, United States; Wayne State University, Detroit, Michigan, United States; University of Detoit Mercy Dental School, Detroit, Michigan, United States; University of Detroit Mercy, Detoit, Michigan, United States; University of Detroit Mercy School of Dentistry, Detroit, Michigan, United States; University of Detroit Mercy School of Dentistry, Detroit, Michigan, United States; University of Detroit Mercy School of Dentistry, Detroit, Michigan, United States

## Abstract

Oral health disparities are widespread among older and disadvantaged Detroit residents. While preventable, poor oral health is strongly associated with adverse health and social outcomes and increased risk of cognitive impairments. To address limitations in access and knowledge among older Detroit persons, the University of Detroit Mercy Oral Health Equity project is providing free drop-in clinics offering screenings and basic care at multiple local community centers. However, we need person-centered knowledge about the reasons for an individual’s current oral health status and to inform strategies to increase service uptake. We conducted interviews with attendees onsite after their services to learn oral health perceptions, wants, and practices. Findings are reported from a sample, n=62, of predominantly African American, median age 81, female 69%, 50% over two years without a dental visit, and 61% fair to poor self-reported oral health. Analyses used topic, theme, pattern, and critical case content analytic methods. Constructs comprising four key domains were identified: (a) Oral health care exists at the intersection of forces featuring societal, political, historical, local urban place, and economic dimensions beyond individual-level health and behaviors; (b) lifespan pathways into current oral health and care practices, including wishes for future oral health status; (c) barriers and resources to oral health care access and practices; and (d) personal knowledge gaps regarding signs and mechanisms of oral health. Discussion will focus on strategies to reduce disparities and increase utilization, community and person-level knowledge gaps, and training dental students to engage older disadvantaged persons effectively.

